# Synthesis of Lead-Free CaTiO_3_ Oxide Perovskite Film through Solution Combustion Method and Its Thickness-Dependent Hysteresis Behaviors within 100 mV Operation

**DOI:** 10.3390/molecules26185446

**Published:** 2021-09-07

**Authors:** Subin Lee, Soyeon Kwak, Taehyun Park, Byoungchul Son, Hyung Joong Yun, Jaehyun Hur, Hocheon Yoo

**Affiliations:** 1 Department of Electronic Engineering, Gachon University, Seongnam 13120, Korea; bini0211@gachon.ac.kr (S.L.); so95yeon@gachon.ac.kr (S.K.); 2 Department of Chemical and Biological Engineering, Gachon University, Seongnam 13120, Korea; thpark@gachon.ac.kr; 3 Research Center for Materials Analysis, Korea Basic Science Institute (KBSI), Daejeon 34133, Korea; sonbc@kbsi.re.kr (B.S.); hjyun@kbsi.re.kr (H.J.Y.)

**Keywords:** perovskite, CaTiO_3_, hysteresis, lead-free, orthorhombic, low voltage, oxygen vacancy

## Abstract

Perovskite is attracting considerable interest because of its excellent semiconducting properties and optoelectronic performance. In particular, lead perovskites have been used extensively in photovoltaic, photodetectors, thin-film transistors, and various electronic applications. On the other hand, the elimination of lead is essential because of its strong toxicity. This paper reports the synthesis of lead-free calcium titanate perovskite (CaTiO_3_) using a solution-processed combustion method. The chemical and morphological properties of CaTiO_3_ were examined as a function of its thickness by scanning electron microscopy, X-ray diffraction (XRD), atomic force microscopy, X-ray photoelectron spectroscopy, and ultraviolet–visible spectrophotometry. The analysis showed that thicker films formed by a cumulative coating result in larger grains and more oxygen vacancies. Furthermore, thickness-dependent hysteresis behaviors were examined by fabricating a metal-CaTiO_3_-metal structure. The electrical hysteresis could be controlled over an extremely low voltage operation, as low as 100 mV, by varying the grain size and oxygen vacancies.

## 1. Introduction

Perovskite is a unique material structure generally expressed as ABX_3_ (where A and B are cations, and X is an anion) that has attracted attraction for its interesting properties, such as ferroelectricity [1], ferromagnetism [2], superconductivity [3], charge ordering [4], and spin-dependent charge transport [5,6,7]. Moreover, perovskite materials achieve their superior optoelectrical performance through a combination of A, B, and X ions. For example, methylammonium lead halide, an inorganic-organic hybrid composite, has been in the spotlight for its overwhelming performance in light-harvesting and photovoltaic research fields and has recently become prominent in a wide range of application areas, including light-emitting diodes [8,9], transistor [10], electronic memory devices [11], and solar cell [12,13,14,15,16,17,18].

Nevertheless, there are still challenges. Lead halide perovskites, the most widely used over the whole applications, suffer from poor stability with high temperature and environmental humidity [19]. In addition, the more severe problem is its toxicity because of its lead content. Exposure to lead can cause serious diseases, such as blindness in parts of the visual field, pregnancy complications, kidney failure, and brain disease [20,21,22]. Therefore, it is important to develop lead-free perovskite materials with an eco-friendly approach.

Oxide perovskites using oxygen as the X anion exhibit superior chemical and thermal stability compared to halide perovskites [23]. Moreover, rich electronic phases, metal-insulator transition, and nonstoichiometric oxygen provide new opportunities in excellent resistive switching behavior using oxide perovskites [24]. Among the various methodologies for thin film deposition, solution-based metal oxide thin film synthesis has attracted considerable attention for its cost-effectiveness, tunable chemical element, and controllable morphology. As an alternative, the synthesis of ternary oxides using self-energy generating combustion chemistry is emerging, and various oxide perovskites have been reported using this technique. Among the previously reported oxide perovskites, calcium titanate (CaTiO_3_, CTO) has been investigated in biomedical [25,26], nanophosphor [27,28], and photocatalytic applications [29,30] because of its excellent characteristics, including innocuous to the human body, high dielectric properties, and permittivity [31,32]. Nevertheless, studies of the thin-film characteristics of CTO depending on the thickness control are insufficient.

In this context, this paper reports the synthesis of lead-free oxide perovskite CTO thin films using a sol–gel combustion spin-coating process, enabling a facile film process of cumulative spin coating. Different from the conventional sol–gel process, we synthesize the CTO thin films by means of the combustion methods. The combustion synthesis is based on redox reaction where the fuel is oxidized, and the oxidizer is reduced leading to an exothermic reaction. Furthermore, we perform comprehensive analysis of the synthesized CTO film by using scanning electron microscopy (SEM), atomic force microscopy (AFM), X-ray photoelectron spectroscopy (XPS), X-ray diffraction (XRD), and ultraviolet–visible spectrophotometry. Depending on the CTO thickness controlled by continuous cumulative spin-coating, thicker films by a cumulative coating results in larger grains and more oxygen vacancies. The I–V electrical characteristics including hysteresis behavior of the metal–CTO film–metal devices were investigated as a function of the thickness. In the case of an enlarged grain size with a higher degree of oxygen vacancies, hysteresis-switching behavior was observed within voltages as low as 100 mV.

## 2. Material and Methods

### 2.1. Materials

Citric acid (99.5%), calcium nitrate tetrahydrate (97%, Ca(NO_3_)_2_·4H_2_O), ethyl alcohol (anhydrous, 99%), isopropyl alcohol (anhydrous, 99.5%, IPA, Seosan, Korea), and titanium isopropoxide (97%, Ti[OCH(CH_3_)_2_]_4_) were purchased from Sigma-Aldrich (St. Louis, MO, USA). Acetic acid (99.5%, CH_3_CO_2_H) and nitric acid (60%, HNO_3_) were supplied from Duksan (Ansan-si, Korea) and Samchun (Pyeongtaek-si, Korea). In the synthesis process, calcium nitrate tetrahydrate, titanium isopropoxide, and citric acid were used as the metal element sources and fuel material, respectively. Nitric acid and acetic acid were used to adjust the pH of the precursor solution.

### 2.2. Precursor Solution Preparation and Device Fabrication

The CTO thin films were synthesized using a sol–gel combustion process with two different metal precursor solutions. First, 10 mL of the metal precursor solutions were prepared by adding calcium nitrate and titanium isopropoxide to a citric acid solution with a 0.3 M concentration. DI water and ethanol were used as solvents for the Ca and Ti solutions, respectively. Subsequently, 0.76 mL of nitric acid and acetic acid was dropped into a Ca and Ti precursor solution to prevent the formation of metal hydroxide. The solutions were stirred until they become transparent. A clear calcium precursor solution was mixed with the Ti precursor solution to form a CaTiO_3_ precursor solution. The molar ratio of Ca, Ti, and citric acid were 1:1:6 for the stoichiometric formula of CaTiO_3_. The solutions were aged overnight with constant stirring to stabilize them. The SiO_2_/Si substrates were cleaned with ethanol and IPA in an ultrasonic bath and dried with a N_2_ blowing gun. The top electrode (Ag, 70 nm) and bottom electrode (Au, 70 nm) were deposited by thermal evaporation at 0.8–1.0 Å/s. The switching layer was fabricated by spin-coating 300 μL of a CTO precursor solution at 3000 rpm for 30 sec, followed by annealing in air at 150 °C for 10 min. The spin coating and annealing process were repeated for various coatings. Thin films were defined by the number of coating times (NCs: 1, NCs: 2, and NCs: 3 for one, two and three-times coating cycle) Subsequently, all devices were placed in a furnace in air at 650 °C for 1 h for crystallization. The patterning of the materials was performed using a metal shadow mask. The distance between electrodes was 90 μm.

### 2.3. Characterization and Device Measurement

The morphology of the as-synthesized CTO films was analyzed by AFM (NX-100, Park system), SEM (S-4700, Hitachi, Tokyo, Japan), and optical microscopy (OM, Inverted TI, Nikon, Japan). Crystallographic and elemental information was identified by energy-dispersive X-ray spectroscopy (EDX, coupled with SEM) and XRD (Smartlab, Rigaku, Tokyo, Japan). UV–vis spectrometry (Lambda 750, Perkin Elmer, Akron, OH, USA) was used to verify the optical absorbance characteristics. The chemical bonding states of the CTO film were investigated by XPS (AXIS SUPRA, Kratos. Inc., Manchester, UK) at the Korean Basic Science Institute (KBSI). The XPS source was monochromatic Al *K*_α_ (*hv* = 1486.6 eV), and XPS was performed with 225 W X-ray power for all spectra. For the electrical characterization, the two-terminal I–V measurements were conducted by sweeping the applied DC voltage from 0 V to 100 mV and from 0 V to −100 mV and performed in air at room temperature using a probe station (Keithley 4200A, Tektronix, Inc., Beaverton, OR, USA).

## 3. Results and Discussion

### 3.1. Synthesis Process and Film Characterization

**Figure 1:**
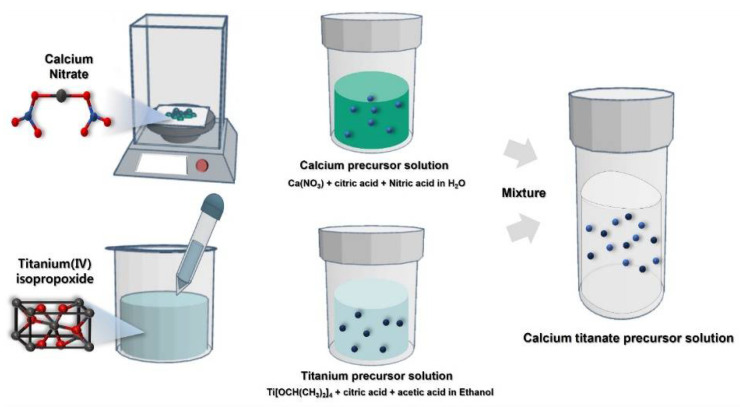
Precursor solution preparation process: the calcium titanate precursor solution was prepared by mixing Ca and Ti solutions at a 1:1 (*v/v*) ratio.

**Figure 2:**
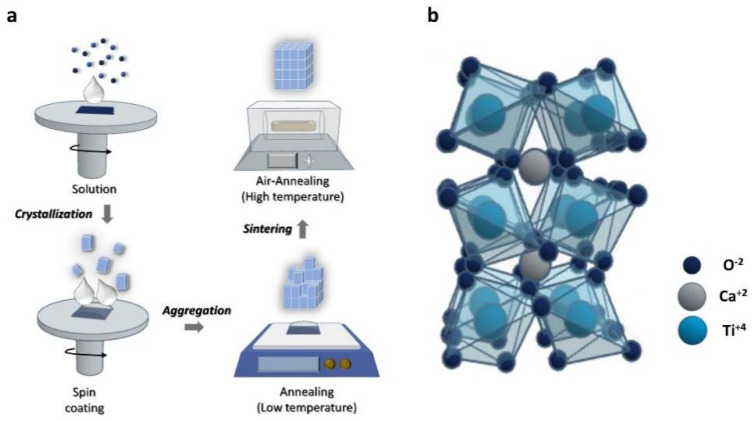
(**a**) Schematic diagram of CTO thin film preparation and (**b**) orthorhombic structure of CTO.

**Figure 3:**
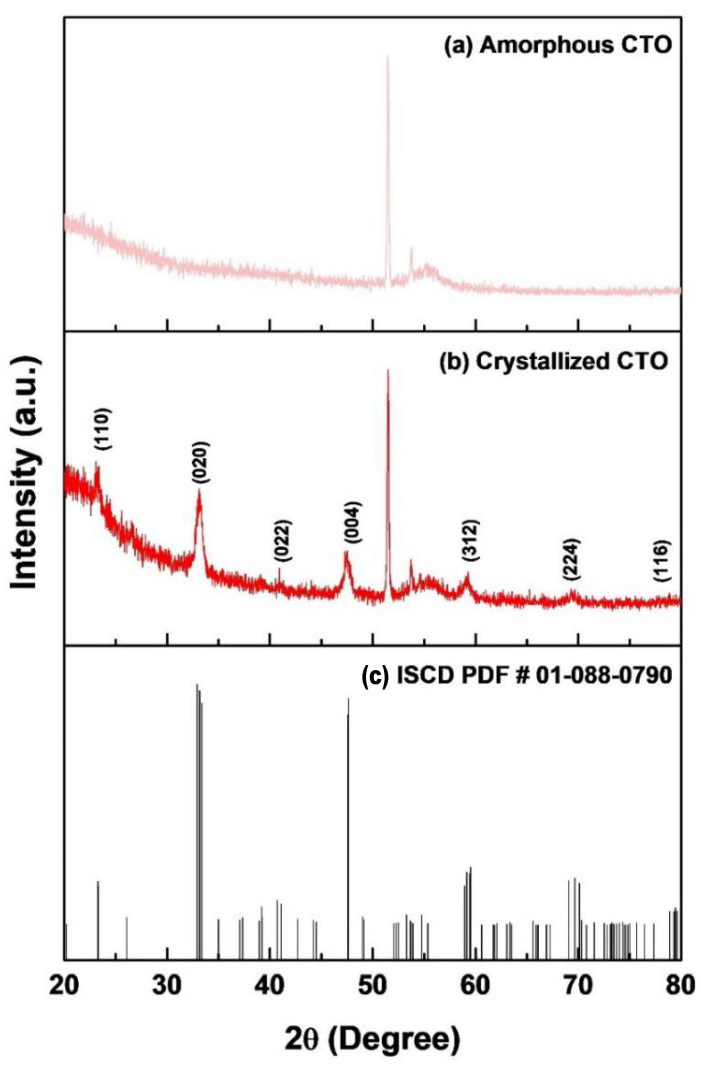
XRD pattern of (**a**) amorphous CTO film, (**b**) crystallized CTO film, and (**c**) the reference for the plane of CTO.

Figure 1 and Figure 2 show a schematic diagram of the CTO thin film synthesis and fabrication process. The combustion reaction mechanism was used to improve the film quality and delicate thickness control. We emphasize that different from the conventional sol–gel synthesis requiring extremely high temperatures, the combustion synthesis uses fuel as an additional energy source by a chemical redox reaction [33]. Various studies achieved high-quality thin films at relatively low crystallization temperatures through combustion synthesis [34,35]. In this study, a soft fuel material (citric acid) was used in the synthesis process. As a result, the fabricated CTO film showed high crystallinity without impurities at low temperatures like to previous studies [36,37]. Detailed crystallographic information is displayed in Figure 3. Figure 3a shows the XRD pattern of an amorphous CTO film sintered at 150 °C. This result suggests that the redox reaction from degrading fuel did not occur under that condition. Under high-temperature annealing condition, fuel could be ignited by the extra energy supplied to metal precursors, resulting in a highly crystallized structure (Figure 3b). The XRD peaks observed at 23°, 33°, 39°, 47°, 59°, 69°, and 79° represent (110), (020), (022), (004), (312), (224), and (116) plane of an orthorhombic perovskite structure (ICSD PDF# 01-088-0790), respectively. Additional peaks at 51°, 53°, and 55° originated from silicon substrate (Figure S1). It is worth noting that side product peaks were not observed in the CTO film, which indicated that the combustion synthesis provided less impurities from precursors.

**Figure 4:**
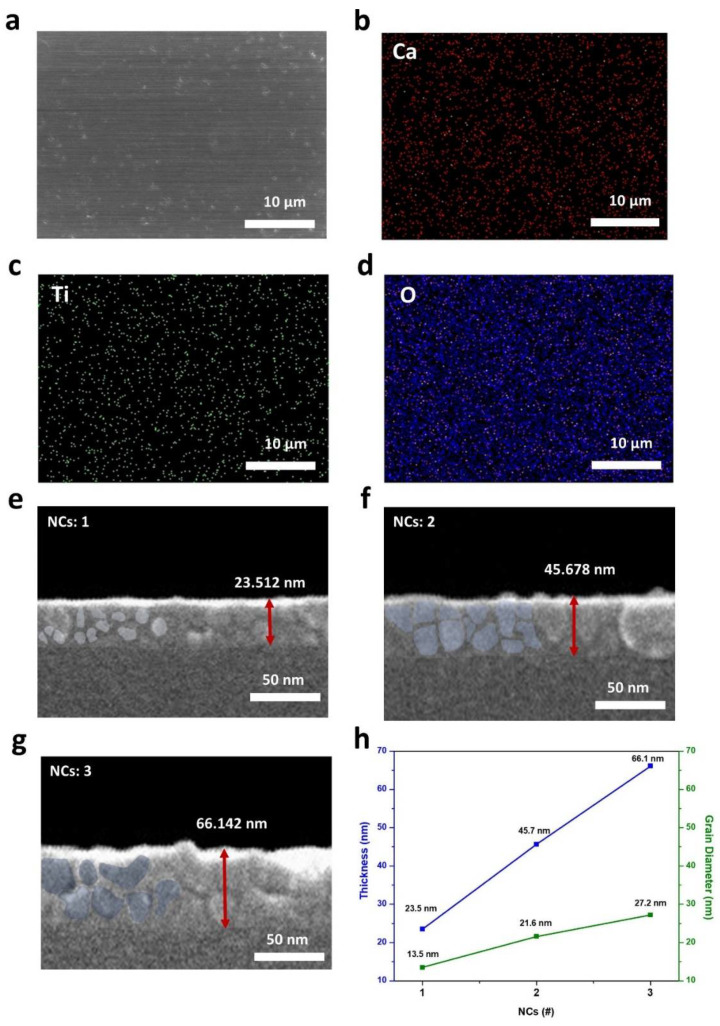
(**a**) Surface SEM image of the CTO film and elemental distribution images of (**b**) Ca, (**c**) Ti, (**d**) O. Cross-sectional SEM images of CTO films varied with the number of coatings where thicknesses are 24.512 nm at (**e**) NCs: 1, 45.678 nm at (**f**) NCs: 2, and 66.142 nm at (**g**) NCs: 3. (**h**) Thickness of the CTO and average diameter of grains under the effect of the number of spin-coating cycles.

**Figure 5:**
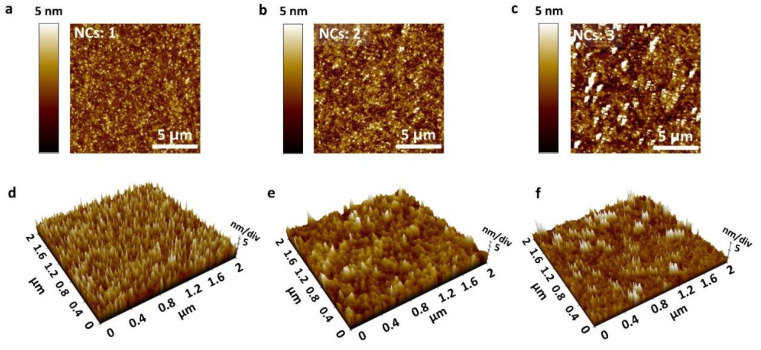
2D (**a**–**c**) and 3D (**d**–**f**) AFM characterization of CTO thin films with different numbers of spin-coating cycles.

Morphological characterization was investigated by SEM (Figure 4) and AFM (Figure 5). EDS mapping revealed a uniform distribution of calcium, titanium, and oxygen (Figure 4a–d). Figure 4e–g show the thickness and grains (light blue colored area) of NCs 1, NCs 2, and NCs 3 films. The thickness of the stacked films exhibited a highly uniform and linear relationship in proportion to the number of coatings. The increased thickness value was approximately 20 nm for each coating process. Generally, in the sol–gel spin coating process, the grain size is affected mainly by the annealing temperature and time, which determine the nucleation growth rate [38]. High external heat energy proceeds with rapid nuclear growth, resulting in films with a large grain size [39]. In this respect, combustion methods using soft fuels and sequential spin coating process promote nucleation and growth due to the internal heat energy from fuel degradation. Figure 4h shows the specific thickness and grain size.

Figure 5a–c,d–f show 2D and 3D AFM images of three different CTO layers. The surface roughness factors, R_a_ (average surface roughness) and R_q_ (root mean squared surface roughness), were extracted from the measurements. The specific values of R_a_ were 0.974, 1.194, and 1.667 nm for NCs: 1, NCs: 2, and NCs: 3. R_q_ values were increased by the number of spin coatings and measured to be 1.263, 1.573, and 2.511 nm, respectively. As shown in the images and parameter values, the increase in surface roughness was insignificant.

**Figure 6:**
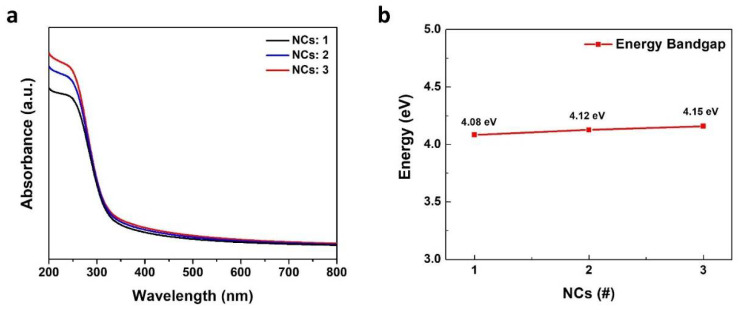
(**a**) UV–vis absorption spectra of NCs: 1, NCs: 2 and NCs: 3, (**b**) optical energy bandgap of NCs: 1, NCs: 2 and NCs: 3.

The optical characteristics of the as-synthesized CTO films were analyzed by UV–vis analysis (Figure 6). The synthesized CTO films showed strong absorption properties in the UVB (280–320 nm) to UVC (200–280 nm) region and high transparency in the visible to long UV range. The absorbance intensity increased with increasing coating cycles, which is in line with the previous thickness increasing tendency. The optical bandgap of the thin films was extracted using the Tauc equation as [40]

(1) $${\left(\alpha h\nu \right)}^{2}=A\left(h\nu -E_{g}\right)$$

where A is a constant; h is the Plank constant; ν is the photon frequency; α is the absorption coefficient of the material; E_g_ is the optical energy bandgap of the material. The optical bandgap of the films increased with increasing film thickness; the specific values were 4.08, 4.12, and 4.15 eV for NCs: 1, NCs: 2, and NCs: 3, respectively. This result suggests that optical band gap of CTO thin film is able to be adjusted by thickness control. The highly transparent property related with its wide band gap imply that CTO thin film is able to be applied as electron transport layer or electron injection layer in optoelectronic research area.

**Figure 7:**
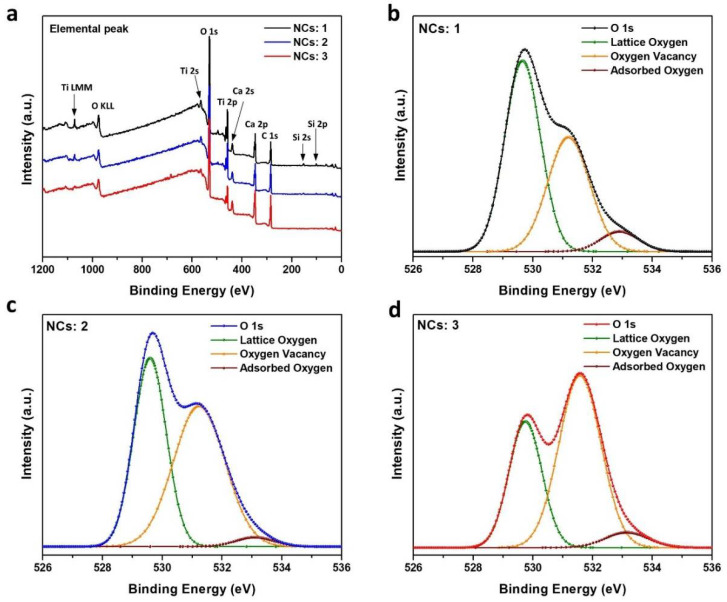
(**a**) XPS photoelectron spectra for three different CTO films. O-1s electronic level of (**b**) NCs: 1, (**c**) NCs: 2, and (**d**) NCs: 3.

XPS of CTO films was carried out to verify the internal and surface chemical bonding state. Figure 7a presents the survey spectrum for the constituent elements. Figure 7b–d shows the spectra corresponding to O-1s for the sample with various thicknesses. In the case of metal oxides, O-1s has three different peaks at 529.7, 531.6, and 532.3 eV, respectively. Each peak corresponds to lattice oxygen, the native defects of O^2−^ vacancies, and chemically adsorbed oxygen species [41]. Table 1 lists the compositions of the three different species obtained from the relative peak area. Interestingly, the peak value related to oxygen vacancies was enhanced by repeated coating process. The fraction of oxygen vacancies for NCs: 1 was 0.38, which increased to 0.51 and 0.60 for the NCs: 2 and NCs: 3, respectively. Figure S2 presents the binding energy peaks of the C-1s, Ti-2p, and Ca-2p states. The peak for C-1s, (Figure S2a) was used to calibrate the charging range at 284.6 eV to fit the lowest binding peak. Ca-2p peak and Ti-2p peak were obtained at 346.7 and 458.1 eV, which is in accordance with previously reported literature [42].

### 3.2. Electrical Characterization of Perovskite Memristor Dependent on CTO Thickness

**Figure 8:**
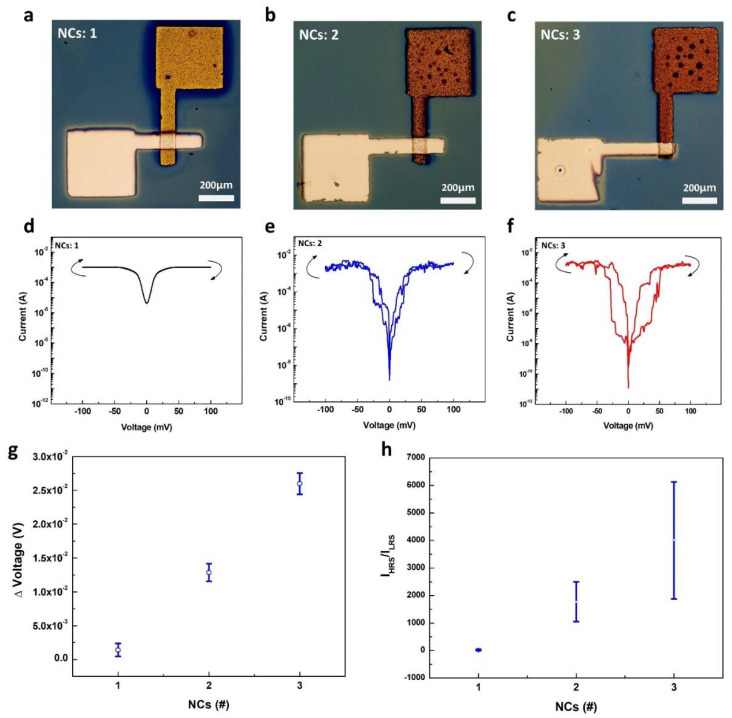
Optical microscopy image of metal-CTO-metal devices varied with the number of coatings (**a**) NCs: 1, (**b**) NCs: 2, (**c**) NCs: 3. I–V curve of metal-CTO-metal devices depending on the number of CTO coatings (**d**) NCs: 1, (**e**) NCs: 2, (**f**) NCs: 3. The plots of the (**g**) memory window and (**h**) switching current ratio.

The set and reset states depend on the values, which are high resistance state (HRS) and low resistance state (LRS). At this time, the voltage applied from the HRS to LRS is called V_set_, and the voltage applied from the LRS to the HRS is called V_reset_. The hysteresis behavior under the effect of the film thickness was investigated by electrical characterization. Electrical hysteresis of the metal–CTO film–metal structured device was observed using current–voltage sweeping measurements. Figure 8a–c shows optical microscope images of devices NCs: 1, NCs: 2, and NCs: 3, respectively. For the device with the NCs: 1 film, no hysteresis was observed over the range of −100 mV < V < 100 mV (Figure 8d). Owing to the thin layer thickness of 23.5 nm, considerable current flow occurred between the top electrode and bottom electrode through the thin CTO, limiting HRS formation. On the other hand, in NCs: 2–3, hysteresis increased as a function of the CTO thickness, which is related directly to the resistance (Figure 8e,f). Five devices consisting of metal-CTO-metal devices were characterized for statistical analysis of the hysteresis behaviors under the effect of the CTO thickness (Figures S3–S5). The plots of the memory window and the switching current ratio are summarized (Figure 8g,h). The hysteresis formation zone could be observed, which is the memory window through the voltage difference (ΔV). The average ΔV of NCs: 1, NCs: 2, and NCs: 3 were 1.42 mV, 12.87 mV, and 25.99 mV and the standard deviation was ±0.96 mV, ±1.3 mV, and ±1.6 mV, respectively. For NCs: 1, the ΔV value was close to 0 mV, indicating no memory window. The average IHRS/ILRS of NCs: 1, NCs: 2, and NCs: 3 were 19.11 A/A, 1775.72 A/A, and 4008.25 A/A and standard deviation were ±27.97 A/A, ±719.96 A/A, and ±2124.70 A/A, respectively, estimated from five samples (Figures S3–S5). These results show that the hysteresis increases as a function of the CTO thickness. The origin of the observed hysteresis can be explained by two phenomena: the grain-boundary-assisted filament formation and enhanced O^2-^ ion filament formation by the enlarged oxygen vacancy fraction. First, the increased grain boundary could be interpreted using the AFM results. According to Figure 5, the number of grains and grain boundaries generated increased with increasing number of CTO coatings [43]. While many grain boundaries in NCs: three allowed the formation of a Ag filament along the boundaries, the fewer grains in NCs: one could not produce such filaments. The current increased rapidly along the filament, and hysteresis occurred. Second, those hysteresis behaviors could have occurred from the migration of O^2−^ ions. When an external electrical field was applied to the films, the charged nonstoichiometric oxygen ions migrate along the electric field direction. The movement of ions leads to local redox reactions, leading to increased local resistivity [44]. XPS verifies the growing oxygen vacancy fraction as the film thickness increased. Therefore, resistive switching behavior could have occurred for the CTO film with more oxygen vacancies (i.e., NCs: 3). Finally, the origin of the low working voltage range could be understood by the characteristics of oxide perovskite. As mentioned earlier, electron-rich phases and a large amount of O^2−^ ions in oxide perovskite materials (e.g., CTO) contribute to increased electric conductivity [24,45].

## 4. Conclusions

This paper reported the facile synthesis of the lead-free oxide perovskite CTO using employing the sol–gel combustion method. By simply controlling the CTO thickness through the cumulative spin coating, this study investigated the morphological and chemical structural changes depending on the film thickness; NCs: 3 showed an enlarged grain structure with more oxygen vacancies than others. As the number of consecutive coatings increased, the grain size with grain boundaries became larger, and the number of oxygen vacancies increased. Furthermore, the electrical properties of the fabricated metal–CTO film–metal structure were analyzed. The results showed that hysteresis appeared within a 100 mV operation level in NCs: 3 because of the formation of grain-boundary-assisted filaments and oxygen vacancy migration. A comprehensive analysis of the thickness-dependent morphology and chemical variations of CTO and its potential use in the extremely low voltage hysteresis behavior was performed. Overall, it is expected that this study offers new opportunities in next-generation lead-free oxide perovskite electronics.

## Figures and Tables

**Table 1:** Relative ratio of the O 1s peak area extracted from XPS.

	Relative Ratio of O 1s Peak Areas		
CTO Film	Lattice	Vacancy	Chemisorb
NCs: 1	0.55	0.38	0.06
NCs: 2	0.43	0.51	0.05
NCs: 3	0.34	0.60	0.05

## Data Availability

Not applicable.

## Supplementary Materials

The following are available online, Figure S1: XRD pattern of P-Si; Figure S2: XPS photoelectron spectra for three different CTO films at each element; Figure S3: I–V curve samples at NCs: 1; Figure S4: I–V curve samples at NCs: 2; Figure S5: I–V curve samples at NCs: 3.
