# Supplementary material for: Synthesis of Lead-Free CaTiO3 Oxide Perovskite Film through Solution Combustion Method and Its Thickness-Dependent Hysteresis Behaviors within 100 mV Operation
Source: Molecules. 2021 Sep 7;26(18):5446. doi: 10.3390/molecules26185446 (PMC8471917; doi:10.3390/molecules26185446)
Supplement: Supplementary file 1 [file molecules-26-05446-s001.zip › molecules-1341606-supplementary.pdf]

molecules

Supplementary Materials: Figure S1: XRD pattern of P-Si, Figure S2: XPS photoelectron spectra for three different CTO films at each element, Figure S3: I-V curve samples at NCs: 1, Figure S4: I-V curve samples at NCs: 2, Figure S5: I-V curve samples at NCs: 3**.**

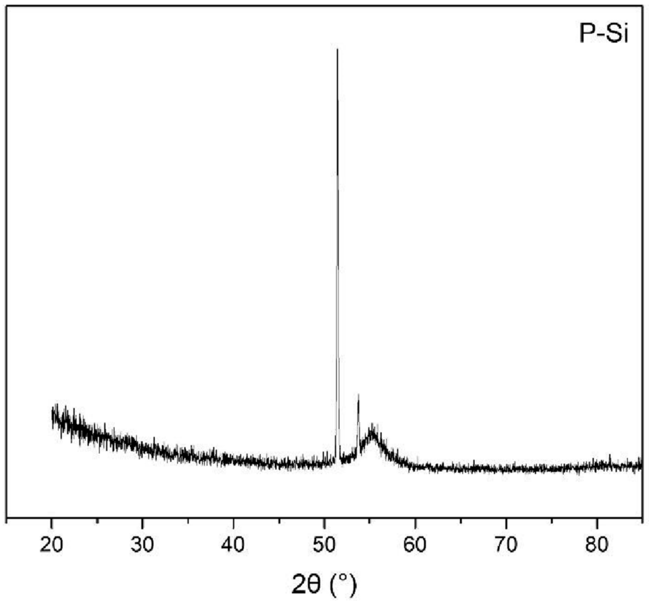

Figure S1. XRD pattern of P-Si. The P-Si peak was measured at all measured patterns: crystallized CTO and amorphous CTO, except for the reference pattern.

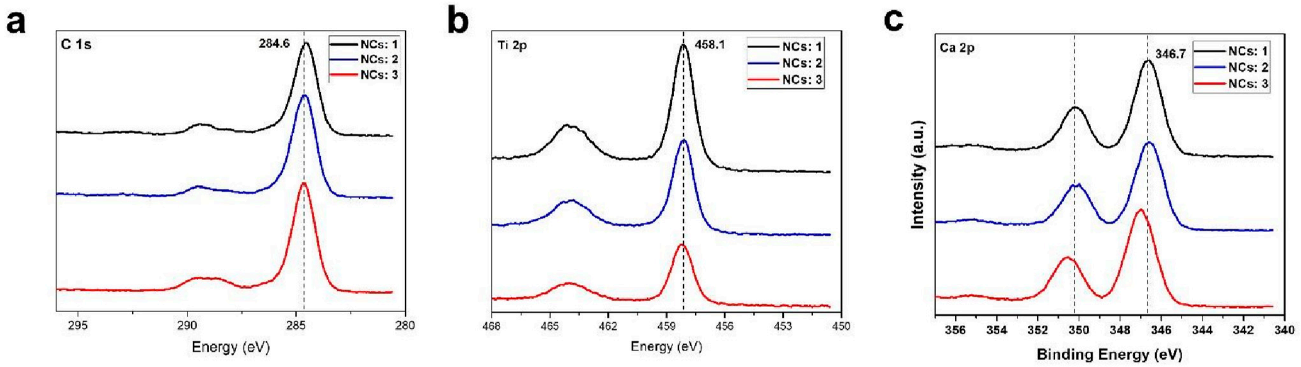

Figure S2. XPS photoelectron spectra for three different CTO films at each element. All three peaks are at the electronic level. (a) The peak of C is for measuring the calibration, and the charging range was 284.6 eV to fit at the lowest binding peak. (b), (c) are the spectra of Ti and Ca, in which the main peaks are at 458.1 eV and 346.7 eV.

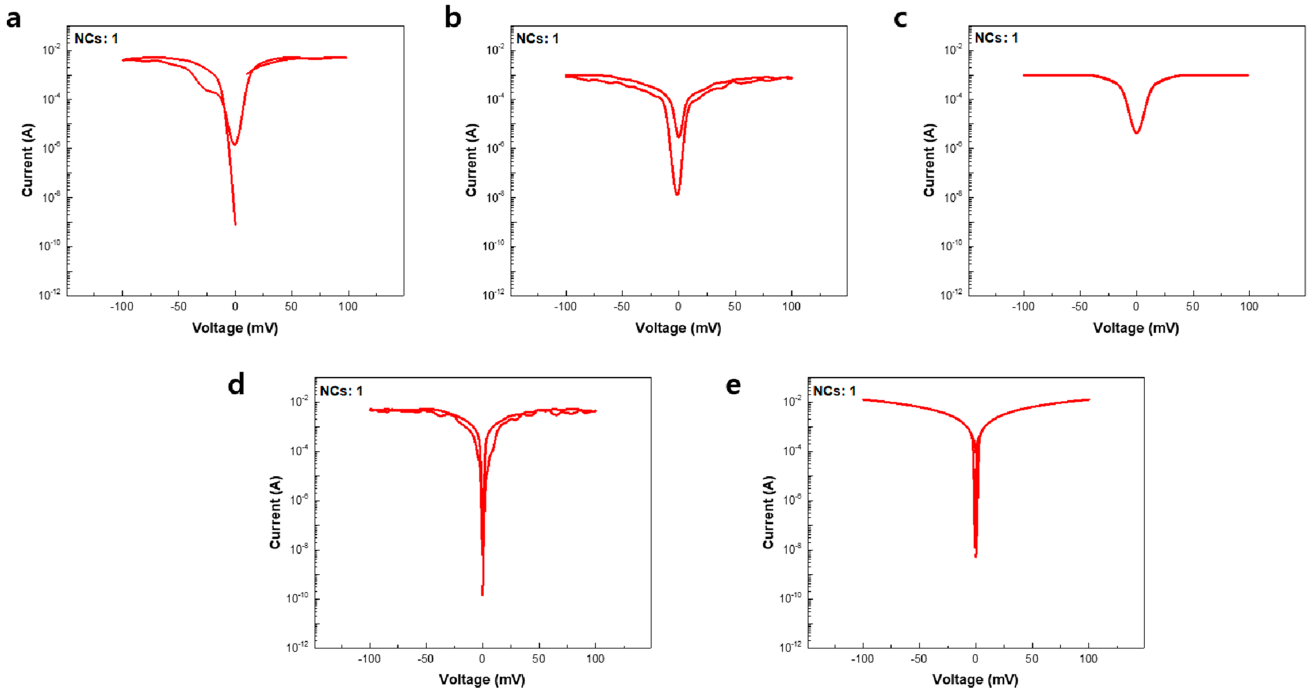

Figure S3. I-V curve samples at NCs: 1, which were used for analyzing △V and I_{HRS}/I_{LRS}. .

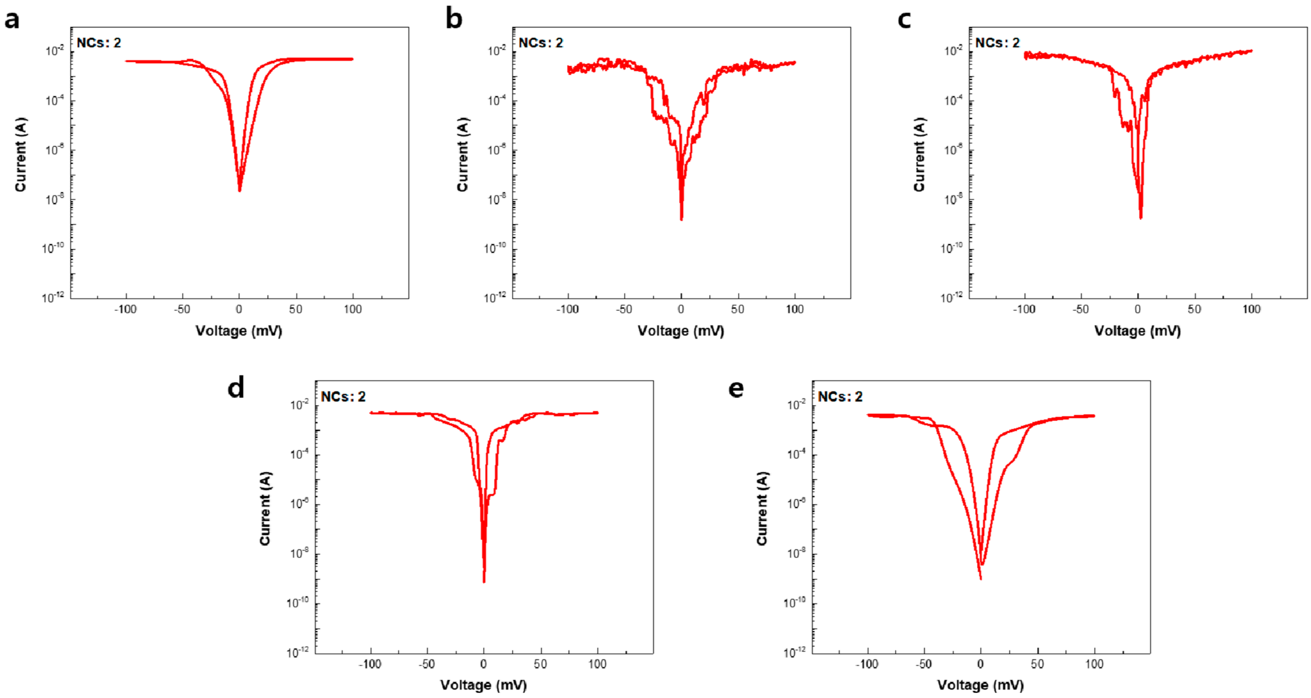

Figure S4. I-V curve samples at NCs: 2, which were used for analyzing △V and I_{HRS}/I_{LRS}. .

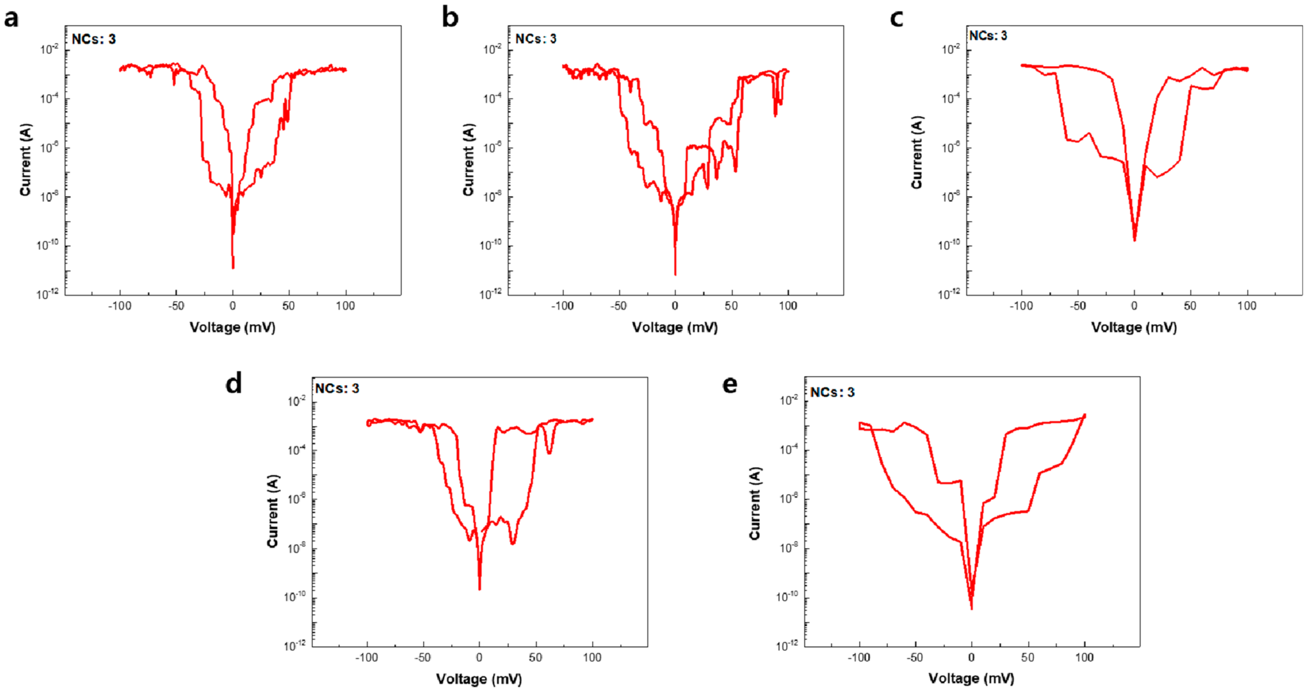

Figure S5. I-V curve samples at NCs: 3, which were used for analyzing △V and I_{HRS}/I_{LRS}.
